# Role of Chlorogenic Acids in Controlling Oxidative and Inflammatory Stress Conditions

**DOI:** 10.3390/nu8010016

**Published:** 2015-12-28

**Authors:** Ningjian Liang, David D. Kitts

**Affiliations:** Departments of Food, Nutrition and Health, the University of British Columbia, Vancouver, BC V6T-1Z4, Canada; ningjian.liang@alumni.ubc.ca

**Keywords:** chlorogenic acid isomers, coffee, antioxidant activity, oxidative stress, anti-inflammation, inflammatory stress

## Abstract

Chlorogenic acids (CGAs) are esters formed between caffeic and quinic acids, and represent an abundant group of plant polyphenols present in the human diet. CGAs have different subgroups that include caffeoylquinic, *p*-coumaroylquinic, and feruloyquinic acids. Results of epidemiological studies suggest that the consumption of beverages such as coffee, tea, wine, different herbal infusions, and also some fruit juices are linked to reduced risks of developing different chronic diseases. These beverages contain CGAs present in different concentrations and isomeric mixtures. The underlying mechanism(s) for specific health benefits attributed to CGAs involves mitigating oxidative stress, and hence the related adverse effects associated with an unbalanced intracellular redox state. There is also evidence to show that CGAs exhibit anti-inflammatory activities by modulating a number of important metabolic pathways. This review will focus on three specific aspects of the relevance of CGAs in coffee beverages; namely: (1) the relative composition of different CGA isomers present in coffee beverages; (2) analysis of *in vitro* and *in vivo* evidence that CGAs and individual isomers can mitigate oxidative and inflammatory stresses; and (3) description of the molecular mechanisms that have a key role in the cell signaling activity that underlines important functions.

## 1. Introduction

**Figure 1:**
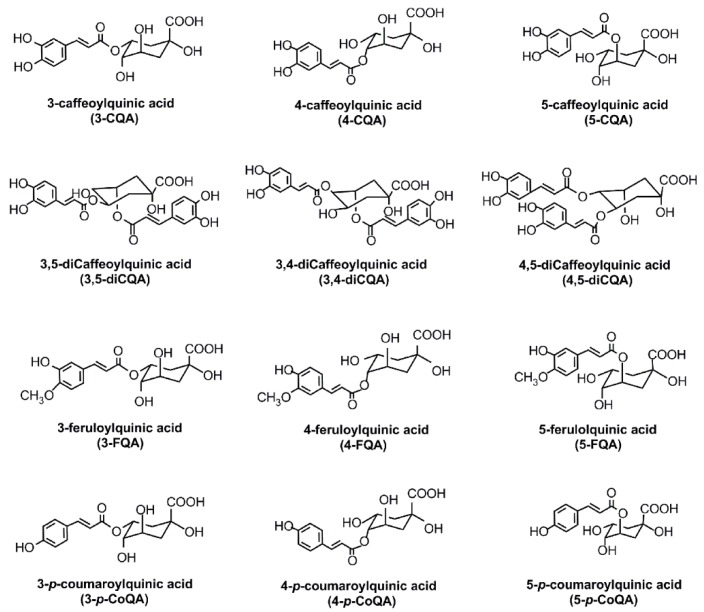
Chemical structures of 3-CQA, 4-CQA, 5-CQA, 3,4-diCQA, 3,5-diCQA, 4,5-diCQA, 3-FQA, 4-FQA, 5-FQA, 3-*p*-CoQA, 4-p-CoQA, and 5-*p*-CoQA.

CGAs are phenolic acids with vicinal hydroxyl groups on aromatic residues that are derived from esterification of cinnamic acids, including caffeic, ferulic and *p*-coumaric acids with quinic acid. A number of conjugated structures, such as caffeoylquinic acids (CQA), dicaffeoylquinic acids (di-CQA), feruloylquinic acids (FQA), and *p*-coumaroylquinic acids (*p*-CoQA), exist in several isomeric forms in coffee beans. Coffee arguably is one of the most popular consumed beverages in the world and is also a very rich source of CGAs. The major CGAs in coffee include 3-caffeoylquinic acid (3-CQA), 4-caffeoylquinic acid (4-CQA), 5-caffeoylquinic acid (5-CQA), 3,4-dicaffeoylquinic acid (3,4-diCQA), 3,5-dicaffeoylquinic acid (3,5-diCQA), and 4,5-dicaffeoylquinic acid (4,5-diCQA). Additional, minor CGAs including 3-feruloylquinic acid (3-FQA), 4-feruloylquinic acid (4-FQA), 5-feruloylquinic acid (5-FQA), 3-p-coumaroylquinic acid (3-*p*-CoQA), 4-p-coumaroylquinic acid (4-*p*-CoQA), and 5-*p*-coumaroylquinic acid (5-*p*-CoQA) are also present in traceable amounts in coffee beverages [1]. CGA lactones are also present after primary thermal processing [2]. The chemical structures of major CGAs are shown in Figure 1. Besides coffee, CGAs are also present widely in beverages prepared from herbs, fruits (e.g., apples, pears, many berries), and vegetables, but consumption from these sources is 5% to 10% of that found from coffee beverage consumption [3]. The health benefits of consuming coffee, tea, fruit juice, and vegetable juice referred to in many epidemiological studies may be linked at least in part to the presence of CGAs in these food systems. There is considerable evidence available to show that CGAs exhibit many biological properties, including antibacterial, antioxidant, and anti-inflammatory activities. This review summarizes the known CGA isomer composition present in different beverages and discusses recent developments that point to potential health benefits that are linked to CGA antioxidant and anti-inflammatory attributes following consumption.

## 2. CGAs Content in Coffee Beans and Coffee Brew

CGA isomer composition in green coffee beans is complex and varies in part according to a number of factors that include the specific coffee variety, the geographic location where it is grown, and the processes used in post-harvest, washing/drying procedures; all of which precedes roasting of the coffee beans. The most abundant CGA in green coffee beans is 5-CQA, which accounts for 76%–84% of the total CGAs, or approximately 10 g/100 g coffee beans [4,5]. Clifford and Ramirez-Martinez [6] measured the CGA composition in two major coffee plant species and reported that green *Coffea Robusta* beans grown in Santos and Sao Paulo contained a higher content of CGAs compared to green *Coffea arabica* beans grown in Ghana and Uganda. Later research has confirmed this finding [7]. For example, Perrone *et al.* [8] reported that total content of CGAs present in green *Coffea canephora* beans grown at Conillon was 86 milligram per gram of dry weight, whereas the total CGA contents in green *Coffea arabica* beans grown at Mundo Novo and Catuai Vermelho ranged from 55 to 63 to milligram per gram of dry weight. Farah *et al.* [2] reported that the total CGAs content in green *Coffea Robusta* beans was 28% higher than the average. In addition to 5-CQA, green coffee beans also contain 3- and 4-CGA, dicaffeolyquinic acids (3,4-, 3,5-, and 4,5-diCQA), feruloylquinic acids (3-, 4-, and 5-FQA), and *p*-coumaroylquinic (3-*p*-, 4-*p*-, and 5-*p*-CoQA) acids [9].

Roasting conditions significantly affect the total CGAs content and profile in processed coffee beans. High-temperature roasting will convert some CGAs into flavor and aroma compounds, or alternatively react with other chemical components in the coffee bean or brew through at least five distinct reaction pathways: namely, epimerization, decarboxylation, acyl migration, lactonization, and dehydration [10]. The final product contributes to melanoidin production in the coffee brew [11]. The idea that CGAs are a component of Maillard reaction end products, such as melanoidin products was initially based on findings that used the Folin–Ciocalteu method for quantifying total phenolics [12]. This has since been confirmed with quantitative analysis of phenolic derivatives recovered from high molecular weight components isolated from coffee brews [13]. Many, if not all, of these reactions that lead to thermal degradation of CGAs during coffee roasting are dependent on the intensity (e.g., time and temperature) of roasting. The diverse and complex nature of products produced with roasting coffee beans varys from relatively simple decarboxylation of quinic and cinnamic acids for simpler phenolic acids, to more complex formation of chlorogenic lactones, derived from dehydration of the quinic acid moiety. The latter evokes intermolecular ester bond formation when CGA is exposed to high heat treatment. Nucleophilic amine- and thio- groups from peptides are also examples of conjugate additions involving CGAs during heating coffee [10]. Hence, the more the coffee beans are roasted, the lower the content of total CGAs. Moon *et al.* [14] reported that around 45%–54% of CGAs were lost in the light roasted (230 °C, 12 min) beans compared to the green beans, whereas more than 99% of CGAs were lost in higher roasts (e.g., city roast, 250 °C for 17 min; French roast, 250 °C for 21 min). Although as much as 99% of CGA could be lost with the greatest roasting, 5-CQA still remained the predominant CGA isomer in roasted coffee. It is interesting that isomers 4-CQA and 3-CQA increased in some varieties of light roasted coffee beans, which could be attributed to isomerization of CGAs brought on by milder heat treatment [14]. The CGA isomer content in roasted coffee beans has been characterized in decreasing order to be: CQA > diCQA > FQA > *p*-CoQA [8]. CGAs composition in a coffee brew prepared from coffee beans with different roasting degrees has also been comprehensively studied. Total CGAs range from 187.7 to 295.6 mg/100 mL brew when prepared from light roasted coffee beans and from 24.2 to 41.3 mg/100 mL brew when prepared from dark roasted coffee beans [15]. Another study reported that the total CGAs in espresso coffee made from light, medium, and dark roasted coffee beans was 1060 mg/100 mL, 517 mg/100 mL, and 340 mg/100 mL, respectively [16]. Regardless of the roasting degree, the content of the total CGAs in espresso coffee from different sources ranged from 89 mg/100 mL to 811 mg/100 mL [17]. The CGA isomer contents in a commercial coffee brew also decreased in the following order, CQA > FQA > diCQA [18,19], which is different from the order reported in roasted coffee beans [8].

The specific procedures used to brew coffee beverages also affect the final content of CGAs, since many factors influence the efficiency of elution of CGAs from ground roasted coffee beans. Filtered coffee is the most widely consumed coffee brew, prepared by pouring boiled water over ground coffee beans that are stationary on a paper filter. In contrast, espresso coffeemakers apply high pressure to force a small amount of boiling water through ground coffee beans. The simplest way of making a coffee brew is by pouring boiling water over the ground coffee beans and waiting for the grounds to settle. All methods of brewing that vary in the ratio between the hot water and ground coffee beans (*v*/*w*), the turbulence, pressure, and the contact surface and contact time will produce a collective effect on the final CGAs profile. Tfouni *et al.* [15] reported that brews prepared by boiling water without filtration had a higher content of CGAs than the corresponding filtered ones. This result might be due to the greater contact of surfaces between the added water and the ground coffee when simply boiled, compared to the filtered method. Ludwig *et al.* [20] compared the CGA isomer composition in espresso coffee and filtered coffee and found that the espresso coffee brew contained relatively more CGAs compared to filtered coffee. Strong pressure applied in espresso favors the extraction efficiency of CGAs into this brew.

## 3. Chlorogenic Acid Content in Other Plant Sources

In addition to green and processed coffee beans being major sources of dietary CGAs, this group of phenolic compounds is also present in fruits and vegetables; again, 5-CQA is the predominant isomer. Fresh potatoes contain CGAs that range from 0.10 to 0.19 mg of 5-CGA per 100 g potato [21], which is equivalent to 90% of the total phenolic compounds present in potato tubers [22,23]. 5-CQA, 5-FQA, and 3,5-diCQA 3,4-diCQA were also detected in different varieties of the vegetable *Chicorium endivia* [24]. Genetically modified tomatoes with increased CGA content have also been developed to enhance antioxidant properties [25]. Popular citrus fruits such as pears and apples are additional rich sources of CGAs. The content of 5-CQA in pears ranged from 0.02 to 3.72 mg per gram of fresh fruit depending on the ripeness of the fruit [26] and type of cultivar [27]. Apples are a rich source of CGAs with the core part having the highest level (2.10 mg per gram of dry fruit), followed by the apple seed (1.10 mg per gram of dry fruit) and then apple flesh (0.48 mg per gram of dry fruit) [28]. CGAs are also present in some herbs. Wang *et al.* [29] studied the CGA profiles in beverages prepared from chrysanthemum, purple sweet potato stem, kuding tea, and honeysuckle flower and reported that 5-CQA and 3,5-diCQA were the dominant isomers with 3-CQA, 4-CQA, 3-FQA, 4-FQA, 5-FQA, 3,4-diCQA, and 4,5-diCQA relatively minor isomers in these beverages. CGAs are the main phenolic compounds in the tea infusions prepared from the herb *Artemisia annua* [30]. In summary, CGAs are widely present in the plant kingdom; many of these plants are important in the human diet. Beverages prepared from coffee beans, fruits, vegetables, and herbs constitute important dietary sources of CGAs. It is interesting to note that the “di-CGA” may contribute different taste qualities, such as producing the bitter/metallic taste found in certain coffees. The significance of this in respect to the taste profile of coffee could be particularly relevant to Robusta coffees, or blends of coffees that contain a proportion of Robusta beans and hence higher amounts of di-CGA.

## 4. Bioavailability and Metabolism of CGAs

**Figure 2:**
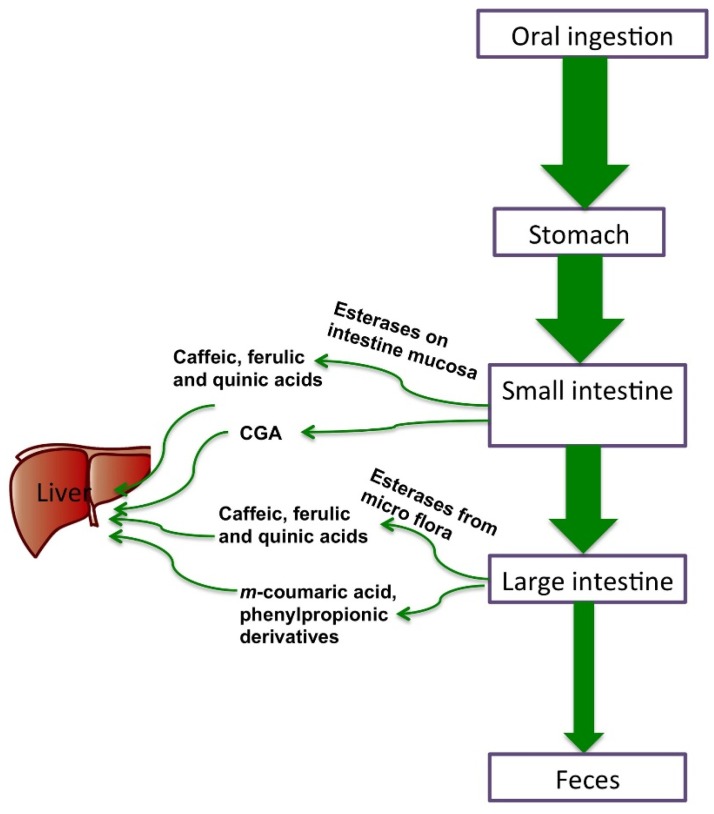
Absorption of CGA when passing through human digestive tract.

In a cultured gastric epithelial model, multiple CGA isomers showed intact transfer across the gastric barrier at an acidic apical pH, with di-CQA having a relatively higher permeability coefficient compared to CQA [31]. Experiments conducted in a rat model showed that CGAs are not hydrolyzed in the stomach but are absorbed in an intact form [32]. These findings could explain the early detection of CGA in plasma within 30 min after coffee consumption. The intestinal absorption of 5-CQA has also been studied in cell culture, using the human colon carcinoma cell line Caco-2, to model the intestinal epithelium *in vitro*. The absorption rate for 5-CQA was 0.10% ± 0.08% at physiological concentrations, or equivalent to concentrations present in gut lumen (0.1~1 mM) [33,34]. Transepithelial transport experiments conducted with CGA using a Caco-2 intestine epithelia cultured monolayer [35] described bidirectional permeation with no transport into the basolaterial side (e.g., 99% retained on apical side), regardless of pH gradient. The permeation rate was concentration-dependent and not saturable, thus indicating passive diffusion. In addition, gut transport was inversely correlated with transepithelial electrical resistance, indicating limited passive diffusion when intestinal junctions are tight. Similar results were observed with caffeic acid, which is absorbed both from paracellular diffusion as well by a monocarboxylic acid transporter (MCT). Strong evidence exists that the majority of CGA is not absorbed in the proximal part of the gastrointestinal tract, unless transformed to caffeic and ferulic acids prior to absorption [32]. Ferulic acid is more efficiently absorbed than CGA, having a monoanionic carboxyl group and non-polar side chain or aromatic hydrophobic moiety that works well with MCT. This may not be the same for CGA, with its noted ester group likely interfering with MCT. With subsequent activity of esterases in both the small intestine mucosa and also microbial esterases in the large intestine, respectively, transformation of CGA to caffeic acid first would occur before further transformation to *m*-coumaric acid and phenylpropionic derivatives [36,37,38]. These main metabolites of CGA are also transported across the intestine cell by MCT. Figure 2 shows the absorption of CGA when passing through human digestive tract. Digestion–balance studies conducted in rats reported that around 9.2% ± 6.8% of CGAs was recovered in the urine 24 h after consumption of a 50 mg/kg dose of CGAs [39]. Some of the original CGA dose was recovered as simpler phenolics, such as caffeic and ferulic acids. Farah *et al.* [9] studied the pharmacokinetic profile and bioavailability of CGAs in healthy human subjects and found that the apparent bioavailability of CGAs from a green coffee extract was 33% ± 23%. Recovery was principally derived from CQA and diCQA, with poor absorption from FQA. Urine was not the major route for excretion of CGAs in the human trial, but smaller metabolites were recovered that suggested the metabolism and excretion of CGA, which influences the overall elimination kinetics in humans, could be quite variable and related to genetic polymorphisms. A similar result (33% ± 17% CGAs) was reported in ileostomy subjects 24 h after consumption of a high dose (2.8 mmol) of CQAs [40]. Further confirmation of this extent of digestion and absorption of CGA has come from a study where coffee CGAs recovered in plasma were quantified from subjects consuming realistic intakes. A positive dose–response describing the absorption efficiency was dependent on the intake level [41]. The bioavailability of the CGAs of coffee beverages can also be affected by various factors that are external to the dietary source. For examples, milk fat added to a coffee beverage may increase CGAs bioavailability [42] and, moreover, the concentration of CGAs present in coffee will also influence bioavailability of CGAs [43]. At present, the influence of the type of food matrix, which influences CGAs digestion and bioavailability, remains unclear and represents an interesting area for more research on factors that influence bioaccessibility of CGAs and other important dietary polyphenols.

## 5. Antioxidant Activity of CGA Isomers

### 5.1. Overview of Antioxidant and Prooxidant Mechanisms and Assays for Assessing Antioxidant Activity in Vitro and in Vivo

Reactive oxygen species [44] and reactive nitrogen species (RNS) are generated endogenously by mitochondrial respiration and are contributed by exogenous exposure to oxidizing agents including ionizing radiation, heavy metals, and hypoxia [45]. The term ROS/RNS is used to include not only the superoxide anions (O_2_^-^), hydroxyl radicals (·OH), nitric oxide radicals (NO·), and peroxyl radicals (ROO·), but also non-radical oxidants, such as hypochlorous acid (HOCl), singlet oxygen (^1^O_2_), peroxylnitrite (ONOO^−^), and hydrogen peroxide (H_2_O_2_), which are all capable of oxidizing important biomolecules [33]. Antioxidant enzymes required to maintain a healthy redox state include: superoxide dismutase (SOD), catalase, glutathione, glutathione peroxidases, and reductase. Dietary components such as vitamin E, vitamin C, and phenolic compounds also serve as non-enzymatic antioxidants. Together, the endogenous and dietary derived antioxidants constitute our antioxidant defense system. Antioxidant phytochemicals derived from beverages constitute a major amount of dietary antioxidants with potential health benefits. In the case of CGAs, these health benefits are a result of CGAs donating hydrogen atoms to reduce free radicals and to inhibit oxidation reactions. After donating hydrogen atoms, CGAs are oxidized to respective phenoxyl radicals and these phenoxyl radicals are quickly stabilized by resonance stabilization. However, pro-oxidant activity of phytophenolics, such as caffeic acid and CGA, respectively, occur when present in systems containing redox-active metals. The redox cycling of CGA in the presence of oxygen is catalyzed by transition metals, such as Cu and Fe, to form reactive oxygen species that are capable of damaging macromolecules, such as DNA and lipids [46]. The extent of pro-oxidant activity of CGA depends on its metal reducing capacity, sequestering behavior and the oxygen-reducing capacity; the latter of which greatly influences the stability/lifetime of the CGA-derived phenoxyl radicals. Phenoxyl radicals generated from catechol ring-containing phenolics, such as caffeic acid and CGA, in the presence of Cu ions are relatively short-lived radicals, but nevertheless they can result in a pro-oxidant effect, such as DNA single strand breaks. This effect has been shown to be small for this class of polyhydroxyl phenolics, and is relatively stronger with caffeic acid than with CGA. The primary reason for this difference is that caffeic acid is a simpler *p*-coumaric derivative, where CGA is esterified and contains a quinic-carbohydrate moiety. This difference evokes a relatively weaker pro-oxidant effect for CGA compared to caffeic acid. Redox-inactive metals such as aluminum and zinc can also enhance the pro-oxidative effect of CGA by producing a spin-stabilizing effect on the CGA phenoxyl radical. The CGA-phenoxyl radical will then be available to induced lipid peroxidation [47]. This potential pro-oxidant activity occurs under conditions that prolong the lifetime of the phenoxyl radical.

Chemical-based assays, cell-based assays, and animal models have been established to gain different levels of understanding about the antioxidant capacity of CGAs. Mechanisms of antioxidant or pro-oxidant activity can be understood using chemical-based assays, where free radicals are artificially generated to react with tested samples under fixed conditions of time and at defined conditions. At the reaction endpoint, the amount of leftover free radicals is measured to reflect the free radical scavenging capacity of the tested sample. Various chemical reactions have been used to generate free radicals. Xanthine oxidase (XOD) utilizes hypoxanthine or xanthine as a substrate and O_2_ as a cofactor to generate O_2_^-^. A Fenton reaction between ferrous iron and H_2_O_2_ produces ·OH. Sodium nitroprusside breaks down to yield NO· and 2,2′-azobis(2-amidinopropane) dihydrochloride (AAPH) constantly generates ROO· at a physiologically appropriate pH.

A cell-based model offers the potential to account for transcellular and paracellular transport processes defining the absorption, and metabolism of the potential antioxidant molecule. It therefore represents and intermediate test between a purely chemical in vitro assay and an animal, *in vivo*, assay. There are a variety of cell-based *in vitro* models available for studying antioxidant activity of different food components, where chemical or physical stressors are used to induce oxidative stress prior to or during exposure of cells to a potential antioxidant compound. The response is often measured using defined biomarkers of redox status to reflect the antioxidant activity of the antioxidant test compound. A commonly used redox biomarker for quantitating response of cells to oxidative stress is the transcription factor, nuclear factor-E2-related factor 2 (Nrf2). Nrf2 is a member of the basic leucine zipper NF-E2 family and plays an essential role in the antioxidant response element-mediated expression of phase II detoxifying enzymes, including glutathione peroxidase (GPx), glutathione reductase (GR), and superoxide dismutase (SOD) [48]. Genomic DNA integrity is another important biomarker of redox status because reactive species continuously attack DNA structure and cause DNA strand breaks, crosslinks, or sister chromatid exchanges, resulting in oxidative damage of DNA [49]. Furthermore, ROS affect the DNA methylation by oxidizing key enzymes involved in the methylation process [45]. Moreover, ROS easily initiate lipid oxidation *in vitro*, leading to the accumulation of lipid peroxidation products such as hydroperoxides and malondialdehyde (MDA), characteristic components of the first and second stages of lipid oxidation reactions, respectively. In order to gain a more comprehensive understanding about the antioxidant activity of food components, such as polyphenols in general, and CGAs specifically, animal models have been employed to study the affinity of plant phenolic compounds to mitigate oxidative stress. Table 1 is a summary of the results obtained from chemical-based studies that have examined the antioxidant activity of CGAs. Table 2 is a summary of the cell-based and animal studies describing the antioxidant activity of CGAs reviewed in this section. In these studies, the chlorogenic acid referred to pertains mostly to 5-CQA.

### 5.2. Evidence from Chemical-Based Assays

All CGA isomers are potent antioxidants, as they possess one to two aromatic rings linked to hydroxyl groups and the one-electron oxidation product of CGAs formed by the reaction with free radicals is rapidly broken down to non-free radical products [50]. Chemical-based assays show that CGAs have the capacity to scavenge 1,1-diphenyl-2-picrylhydrazyl (DPPH) radicals, superoxide anions (O_2_^-^), hydroxyl radicals (·OH) [51,52], 2,2′-azino-bis(3-ethylbenzothiazoline-6-sulphonic acid) (ABTS) radicals [53], lipid oxidation [53,54], and peroxylnitrite (ONOO^-^) [54]. CGAs react with difference sources of free radicals at different rate constants. The second-order rate constants for 5-CQA reacting with superoxide, hydroxyl radical, peroxyl radical, and peroxynitrite have been determined to be 0.96 ± 0.01 × 10^6^ M^−1^·s^−1^, 3.34 ± 0.19 × 10^9^ M^−1^·s^−1^, 1.28 ± 0.11 × 10^5^ M^−1^·s^−1^, and 1.6 ± 0.7 × 10^5^ M^−1^·s^−1^, respectively [54]. This result shows that the relative efficiency of 5-CQA to react with free radicals is species-specific to the radical. Another important activity of CGAs towards ROS-induced oxidative stress involves the radical damage caused to DNA, which can be quantified by measuring DNA strand breakage. Six CGA isomers, namely 3-CQA, 4-CQA, 5-CQA, 3,5-diCQA, 3,4-diCQA, and 4,5-diCQA, were shown to exhibit a protective effect against H_2_O_2_-induced DNA plasmid chromosome breaks [55]. Secondary byproducts of the oxidation reaction are also relevant in initiating a mutagenic response as a result of DNA damage. One example concerns the oxidation of chloride by H_2_O_2_, resulting in formation of HOCl, and subsequent reaction with amines to produce NH_2_Cl, an oxidant and potent mutagen. CGAs, specifically 5-CQA, have been shown to protect against NH_2_Cl-induced plasmid DNA breakage in cultured neutrophils [50]. Another example of the anti-peroxidation activity of 5-CQA comes from the protection against LDL oxidation, which is the initial step in the development of atherosclerosis. Studies based on incubating isolated LDL with oxidizing agents *in vitro* showed that 5-CQA was effective at mitigating both copper-induced LDL oxidation [56] and ferryl myoglobin-induced LDL oxidation [57]. These results correspond to other studies that reported reduced MDA content in brain tissues when pre-treated with 5-CQA [53].

### 5.3. Evidence from Cell-Based Assays

Research directed at characterizing the antioxidant capacity of CGAs using cell-based models has focused mostly on the 5-CQA isomer. This CGA isomer protects against H_2_O_2_-induced oxidative stress in human HaCaT cells [51]; it can activate Akt phosphorylation, increase the expression of FOXO family genes in mesenchymal stem cells of bone marrow [58], and reduce apoptosis in primary cortical neurons by upregulating antioxidant enzymes such as NADPH:quinine oxidoreductase 1 [59]. The protective effect of 5-CQA against oxidative stress stimulated by various oxidative stressors (e.g., *t*-BHP, H_2_O_2_, FeSO_4_) has also been studied in PC12 cells, which used multiple biomarkers such as the reduction of lipid peroxidation product, the extent of ROS formation, and GSH depletion to evaluate the utility of CGAs to prevent oxidation [60]. 5-CQA showed effectiveness in protecting against DNA damage with activity to inhibit methylation of the promoter region of the RAR beta gene through increased formation of *S*-adenosyl-l-homocysteine in cultured human breast cancer cells MCF-7 and MAD-MB-231, respectively [61]. 5-CQA also decreased the DNA damage by 4.49%~48.15% in human blood lymphocytes caused by X-ray irradiation [62].

In addition to these examples of chemically induced oxidation, other studies have successfully shown that CGAs are effective at reducing the damage caused by exposure to ultraviolet light (UVB) [63]. The photo-oxidation protection offered by 5-CQA in a mouse epidermal cell line [64] and human HaCaT keratinocytes [51] was related to the effects of 5-CQA to trigger induction of Nrf2 transactivation and phase II enzyme activities.

There are only a few studies that have investigated the affinity of minor CGA isomers to modulate redox status in biological systems. Three CGA isomers, namely 3-CQA, 4-CQA, and 5-CQA, have affinity to protect against H_2_O_2_-induced apoptosis in PC12 cells by suppressing the mitochondrial membrane depolarization caused by oxidative stress [65]. Recently, another research group reported that 5-CQA and 3,5-diCQA had a protective effect against *t*-BOOH-induced ROS generation in HepG2 cells [66].

**Table 1:** The antioxidant activity of CGA isomers evaluated by chemical assays.

Chemical Assays	End-Point Measure	CGA Isomer	Concentration/Exposure Time	Results	References
DPPH assay	DPPH	5-CQA	5–80 μM for 3 h	10%~90% inhibition on DPPH	[51]
Xanthine/xanthine oxidase system	DMPO/·OOH adducts	5-CQA	20 μM for 2.5 min	↓ 30% ·OOH	[51]
FeSO_4_ + H_2_O_2_	DMPO/·OH adducts	5-CQA	20 μM for 2.5 min	↓ 51% ·OH	[51]
FeSO_4_ + H_2_O_2_	DMPO/·OH adducts	5-CQA	100–400 μM for 1 min	↓ 50% to 80% ·OH	[52]
ABTS assay	ABTS·^+^	5-CQA	Serials concentration for 15 min	The ability of 100 g of CGA in scavenging ABTS·^+^ is equivalent to 3.7 mmol Trolox	[53]
Rat brain homogenates + sodium nitroprusside	MDA	5-CQA	1.56–6.25 μg/mL	No significant inhibition of MDA	[53]
Liposome system containing AAPH	MDA	5-CQA	0.1–0.5 mM	Second order rate of constant of the reactions of LOO· with CGA is 1.28 ± 0.11 × 10^5^ M^−1^·s^−1^	[54]
Pulse radiolysis to generate O_2_^-^	O_2_^-^	5-CQA	0.2–0.75 mM	Second order rate of constant of the reactions of O_2_- with CGA is 0.96 ± 0.01 × 10^6^ M^−1^·s^−1^	[54]
Fenton-type reaction to generate ·OH	·OH	5-CQA	0.1–0.75 mM	Second order rate of constant of the reactions of ·OH with CGA is 3.34 ± 0.19 × 10^9^ M^−1^·s^−1^	[54]
Potassium phosphate to generate ONOO^-^	ONOO^-^	5-CQA	80 μM	Second order rate of constant of the reactions of ONOO^-^ with CGA is 1.6 ± 0.7 × 10^5^ M^−1^·s^−1^	[54]
DPPH assay	DPPH	3-CQA,	5 μg/mL–60 μg/mL	EC_50_^a^ 3-CQA:13.4 μg/mL	[55]
		4-CQA,		4-CQA: 13.2 μg/mL	
		5-CQA,		5-CQA: 13.8 μg/mL	
		3,5-diCQA,		3,5-diCQA: 9.3 μg/mL	
		3,4-diCQA,		3,4-diCQA: 9.4 μg/mL	
		4,5-diCQA		4,5-diCQA: 7.5 μg/mL	
ABTS assay	ABTS·^+^	3-CQA	50 μg/mL–150 μg/mL	EC_50_^a^ 3-CQA: 91.4 μg/mL	[55]
		4-CQA,		4-CQA: 87.5 μg/mL	
		5-CQA,		5-CQA: 91.5 μg/mL	
		3,5-diCQA,		3,5-diCQA: 77.6 μg/mL	
		3,4-diCQA,		3,4-diCQA: 77.4 μg/mL	
		4,5-diCQA		4,5-diCQA: 67.3 μg/mL	
FRAP assay	Reducing power	3-CQA	25–125 μg/mL	4,5-diCQA > 3,5-diCQA > 3,4-diCQA > 5-CQA = 4-CQA = 3-CQA	[55]
		4-CQA,			
		5-CQA,			
		3,5-diCQA,			
		3,4-diCQA,			
		4,5-diCQA			
DNA damage protective effect assay	DNA damage	3-CQA	50 μg/mL	↓ 43.1 to 62.4% DNA damage	[55]
		4-CQA,			
		5-CQA,			
		3,5-diCQA,		4,5-diCQA > 3,4-diCQA > 3,5-diCQA > 5-CQA > 4-CQA > 3-CQA	
		3,4-diCQA,			
		4,5-diCQA			
Plasmid pUC18 + NH_2_Cl	Supercoiled DNA, nicked circular DNA and linear duple	5-CQA	0.01 mM–1.23 mM	Prevented a stepwise conversion of plasmid DNA form supercoiled DNA, nicked circular DNA and linear duplex DNA	[50]
LDL + copper	Conjugated dienes	5-CQA	0.25–1.0 μM	↑ lag time of LDL oxidation	[56]
LDL + metmyoglobin + H_2_O_2_	ROS	5-CQA	1 molar ratio to metmyoglobin	Effectively blocked LDL oxidation	[57]

^a^ EC_50_ represents the concentration of the tested compound that results in half-maximal response.

**Table 2:** Summary of studies that evaluated the capacity of CGAs to modulate oxidative stress in cell-based and animal-based models.

Model	End-Point Measure	CGA Isomer	Concentration/Exposure Time	Results Compared to the Control without CGA Treatment	References	
**Cell-based assay**						
HaCaT cell + H_2_O_2_	H_2_O_2_	5-CQA	20 μM for 20 h	↓ ROS		[51]
HaCaT cell + UVB	ROS, DNA damage, cell viability	5-CQA	20 μM for 20 h	↓ ROS		[51]
				↓ DNA damage		
				↑ 13% cell viability		
Mesenchymal stem cell + H_2_O_2_	Chromosomal condensation, cell apoptosis, ROS	5-CQA	10 mM for 12 h	↓ Chromosomal condensation		[58]
				↓ Cell apoptosis		
				↓ ROS		
Primary cortical neurons + H_2_O_2_	NADPH: quinine oxido-reductase 1, Cell viability	5-CQA	12.5–100 μM for 1 h	↑ NADPH: quinine oxido-reductase 1		[59]
				↑ Cell viability		
Differentiated neuronal PC12 cells + H_2_O_2_	Cell viability, GSH	5-CQA	6.2–25 μM for 2 h	↑ Cell viability		[60]
				Attenuated GSH decrease		
Differentiated neuronal PC12 cells + FeSO_4_	Cell viability, ROS, MDA	5-CQA	6.2–25 μM for 2 h	Did not change cell viability		[60]
				↓ ROS level		
				↓ MDA		
Differentiated neuronal PC12 cell + *t*-BHP	Cell viability, GSH	5-CQA	6.2–25 μM for 2 h	↑ Cell viability		[60]
				Did not change GSH level		
Human breast cancer cell line MCF-7+	Global methylation status	5-CQA	1–20 μM for 8 days	↓ Global methylation		[61]
Human breast cancer cell line MDA-MB-231+	Global methylation status	5-CQA	0.2–20 μM for 3 days	Did not change the global methylation status		[61]
Human breast cancer cell line T-47D+	Global methylation status	5-CQA	20–50 μM for 2 days	↓ Global methylation		[61]
Human lymphocyte + X-ray radiation	Genetic damage index	5-CQA	0.5–4 μg/mL	↓ Genetic damge index by 4.49% to 48.15%		[62]
Mouse epidermal cell line JB6 + UVB	GST, NADPH: quinone oxido-reductase, Nrf2	5-CQA	5–160 μM for 1 h	↑ GST		[64]
				↑ NADPH:quinone oxido-reductase		
				↑ Nrf2 nuclear translocation		
Differentiated neuronal PC12 cell + H_2_O_2_	Mitochondrial membrane depolarization	3-CQA, 4-CQA, 5-CQA	10 μM for 20 min	Protected mitochondrial membrane depolarization through		[65]
				↓ Caspase 9 activation		
Human hepatoma HepG2 cell + *t*-BOOH	ROS, GSH, GPx, GR, MDA	5-CQA, 3,5-diCQA	10–20 μM for 20 h	↓ ROS, ↑ GSH, ↑ GR, ↓ GPx, ↓ MDA		[66]
**Animal Models**						
Type 2 diabetic rat model	Lipid peroxidation, GSH, V_C_, V_E_	5-CQA	Oral administration at 5 mg/kg body weight daily for 45 days	↓ Plasma lipid hydroperoxides, ↑ GSH, ↑ V_C_, ↑ V_E_		[67]
Type 2 diabetic rat model	Lipid peroxidation, GST, SOD, GPx, CAT	5-CQA	Oral administration at 5 mg/kg body weight daily for 45 days	↓ Lipid oxidation, ↑ GST, ↑ SOD, ↑ GPx, ↑ CAT		[68]
Methamphetamine induced oxidative stress rat model	NO, MDA, SOD, GPx	5-CQA	Oral administration at 60 mg/kg body weight, single dose	↓ NO, ↓ MDA, ↑ SOD, ↑ GPx		[69]
Cd induced brain impairment rat model	SOD, CAT, GPx, GSH, V_C_, V_E_, MDA	5-CQA	Intragastric administration, 60 mg/kg body weight daily for 30 days	↑ SOD, ↑ CAT, ↑ GPx, ↑ GSH, ↑V_C_, ↑ V_E_, ↓ MDA		[70]
Scopolamine induced brain impairment rat model	MDA	5-CQA	Orally administered at 3–9 mg/kg body weight, single dose	↓ MDA		[71]
Benzopyrene induced gastrointestinal pathogenesis rat model	GST, Cytochrome P-450	5-CQA	Eating 0.2% 5-CQA containing diet for 10 weeks	↑ GST,		[72]
				Did not significantly change cytochrome P-450		
Sodium pentobarbital induced intestinal ischemia-reperfusion rat model	Vascular permeability in the small intestine	5-CQA	Directly administrate 1 mM into jejunum, single dose	Attenuated the increased vascular permeability		[73]
Azoxymethane induced colon cancer mice model	GSH/GSSG ratio	5-CQA	Orally administered at 0.1% 5-CQA containing diet for 20 weeks	↑ Hepatic GSH/GSSG ratio		[44]
UV irradiation induced erythema formation in Guinea pig and Yucatan micropig	Erythema	5-CQA	Intradermal delivery of 5-CQA at 1.49 μmol/g skin	Prevented erythema formation induced by UV irradiation		[74]
Gamma irradiation induced chromosomal damage in mice model	Frequencies of micro-nucleated polychromatic erythrocytes	5-CQA	Orally administered at 50–200 mg/kg body weight, single dose	↓ Frequencies of micro-nucleated polychromatic erythrocytes		[75]

### 5.4. Evidence from Animal-Based Assays

Given the positive data from cell culture experiments that show antioxidant properties of specific CGAs at both cellular and molecular levels, other studies conducted in rodent models have confirmed these observations by examining the redox status in animals exposed to a variety of forms of oxidative stress when fed CGAs. Furthermore, the efficacy of dietary intake of CGAs to prevent pathogenesis of a wide range of chronic disease states has been examined. One example includes the side effect of hyperglycemia that occurs with diabetes, and which leads to an increase in ROS production and increased susceptibility to oxidative stress [76]. The antioxidant activity of 5-CQA in diabetic rat models showed that feeding 5-CQA effectively reduced lipid hydroperoxide production and increased the level of non-enzymatic antioxidants such as reduced glutathione and Vitamins C and E [67,68]. Other studies have shown that 5-CQA can alleviate the oxidative stress induced by methamphetamine in rats by restoring liver SOD and GPx activities and preventing the accumulation of MDA [69].

The role of CGAs in prevention of environmental toxicity caused by heavy metal pollution, specifically cadmium (Cd), has produced data showing protection against the induction of oxidative stress in the central nervous system [77]. Pretreatment of rats with 5-CQA prior to Cd exposure significantly restored the depleted levels of GSH, vitamin C, and vitamin E, and attenuated Cd-induced MDA levels in brain tissue [70]. Other model systems used scopolamine, a muscarinic antagonist that significantly increases MDA levels in the cortex and hippocampus [78]. The scopolamine-induced amnesic mouse, an animal model to study Alzheimer’s disease, has produced data to show that 5-CQA decreased the MDA level in both the frontal cortex and the hippocampus of scopolamine-induced anemia in mice [71]. The anti-amnesic activity of 5-CQA was attributed to the affinity to reduce lipid peroxidation in addition to reducing free radical scavenging activity [71]. Former studies have also examined the role of 5-CQA to enhance detoxification of environmentally toxic residues derived from polyaromatic hydrocarbon (PAHs) exposure in mice [72]. Dietary CGAs were effective at enhancing gastrointestinal xenobiotic detoxification enzymes that are central to the detoxification of PAHs. The more recent finding that 5-CQA protects against oxidative stress through its activation of Nrf2 nuclear translocation and upregulation of cellular antioxidant enzymes [64] confirms the observation reported earlier in mice fed PAHs. ROS are also implicated in the development of ischemia/reperfusion (I/R) injury in the intestine [72] and pathogenesis of colorectal cancer. An intestinal I/R model and a colorectal cancer model were used to assess the ability of 5-CQA in alleviating oxidative stress in these sections of the gastrointestinal tract. Sato *et al.* [73] reported in rats with I/R injury induced by sodium pentobarbital, that dietary intake of CGA at concentrations ranging from 0.5 to 1.0 mM was effective to improve the capillary permeability of the small intestine and the capacity to reduce oxidative stress. In an azoxymethane-induced colon cancer mouse model, a 20% reduction in small intestinal GSH levels occurred, pointing to an induced oxidative stress condition. Researchers found that feeding diets containing 0.1% 5-CQA for 20 weeks attenuated azoxymethane-induced oxidative stress by bringing GSH levels back to normal levels [44].

Ultraviolet radiation and gamma radiation can also trigger the generation of ROS and consequently cause chromosomal damage in animals [63]. Intradermal delivery of 5-CQA in guinea pigs during exposure to UVB reduced photooxidation-induced damage of skin attributed to photooxidation stress [74]. Another study showed that oral administration of 5-CQA to mice at a concentration of 100 mg/kg body weight before exposure to gamma radiation significantly reduced chromosomal damage [75].

Since oxidative stress is implicated with a wide range of chronic diseases, it is challenging to understand the role of specific antioxidants in different pathological and physiological conditions. Numerous animal models have been used to identify a useful biomarker that will reflect the initiation of oxidative stress so that the quality of the antioxidant can be evaluated. Despite using different methodologies, there is strong evidence that CGAs are effective antioxidants that will protect against oxidation reactions *in vivo* by up-regulating redox-related nuclear transcription factors involved in the expression of antioxidant enzymes. The majority of the studies are focused on the primary isomer of CGA, 5-CQA, with a lesser amount of information available for other minor CGA isomers.

## 6. The Ability of CGA on Modulating Inflammatory Responses

### 6.1. Overview of Inflammation and Anti-Inflammatory Mechanisms

Inflammation is a physiological response to tissue injury caused by exogenous or endogenous sources. Exogenous inducers include pathogen-associated molecular patterns, virulence factors, allergens, foreign bodies, and toxic compounds [79]. Endogenous inducers of inflammation arise from cell signaling in response to damaged or malfunctioning tissues [80,81]. It is believed that a controlled inflammatory response is required to combat offending agents and result in the return of tissue homeostasis. However, a dysregulated inflammatory response could lead to the failure of effective resolution, thus leading to excessive tissue damage, resulting in acute or chronic disease states [82]. Anti-inflammatory drugs have been developed to resolve conditions of dysregulated inflammation by targeting inflammatory mediators, or modulating the activity of cell signaling cascades involved in responding to an inflammatory signal. The nuclear factor kappa B (NF-κB) pathway is a key regulator of the release of pro-inflammatory cytokines, chemokines, and adhesion molecules [83]. Nonsteroidal anti-inflammatory drugs (NSAIDs) are the most widely used drugs for the treatment of inflammatory diseases [84]. The cyclooxygenase (COX) pathway is the major target for NSAIDs because COX catalyzes fatty acid oxygenation to produce eicosanoids, which are the cardinal signs of inflammation. Side effects of NSAIDs include a predisposition to ulcers and bleeding in the stomach and intestines. Thus, there is increased interest in searching for novel agents that may have anti-inflammatory activity, without inducing adverse side effects. Different cell lines triggered with pathogen-associated molecular patterns (lipopolysaccharide (LPS)) and pro-inflammatory cytokines (e.g., tumor necrosis factor-alpha (TNF-α), interleukin 1β (IL-1β), and interferon gamma (IFN-γ)) have been used as cell-based inflammation models to study anti-inflammatory mechanisms. Also, animal models of inflammatory bowel disease, rheumatoid arthritis, and injury-associated inflammation have been successfully used to evaluate the anti-inflammatory effect of different components, such as dietary bioactives.

### 6.2. CGA Suppression of Inflammation through Inhibition of Pro-Inflammatory Cytokines via Modulation of Key Transcription Factors

Inflammatory bowel disease represents a chronic relapsing of disorders occurring in the gastrointestinal tract that are characterized by intestinal inflammation and epithelial injury [85]. 5-CQA has a protective effect against intestinal-related inflammation in both cell-based and animal-based models. CGA has an anti-inflammatory effect in TNF-α and H_2_O_2_-induced human intestine epithelia Caco-2 cells by down regulating IL-8 production [86]. Studies conducted with a tea (*Artemisia annua*) containing CGAs showed a strong anti-inflammatory effect by decreasing the secretion of pro-inflammatory cytokines IL-8 and IL-6 in Caco-2 cells stimulated with TNF-α, LPS, IL-1β, and IFN-γ [30]. CGA also attenuated IL-1β, TNF-α, and IL-6 production in LPS-stimulated murine RAW 264.7 macrophages and in BV2 microglial cells by effectively down regulating the NF-κB pathway [87]. Previously, it has been reported that phenolic compounds lower the activity of COX and subsequently prevent the synthesis of eicosanoids [88]. A cell study conducted on murine RAW 264.7 macrophages confirmed that CGA showed anti-inflammatory activity by suppressing LPS-induced COX-2 expression via attenuating the activation of NF-κB and JNK/AP-1 signaling pathways [89]. In animal studies, the oral administration of 5-CQA protected against trinitrobenzenesulfonic acid-induced colitis in mice by reducing neutrophil infiltration and inhibition of the NF-κB pathway [48]. A similar effect was also observed in the dextran sulfate sodium-induced colitis model in mice [86] and in a carrageenan-induced paw edema model in rats [90]; in both cases a suppression of pro-inflammatory cytokines was observed.

Rheumatoid arthritis is another chronic inflammatory disorder characterized by the deterioration of cartilage and bone. Chauhan *et al.* [91] observed that oral administration of 40 mg/kg of 5-CQA effectively suppressed pro-inflammatory cytokines including TNF-α and IL-1β in an LPS-induced knee joint inflammation rat model. This effect was similar to the standard treatment of administering ibuprofen at 100 mg/kg.

The reduction of inflammation resulting in enhanced wound healing has also been reported for CGAs. Oral administration of 5-CQA at a dose of 50 mg/kg/day accelerated wound healing and decreased MDA and nitric oxide levels while elevating reduced-glutathione content in rats [92]. Oral administration of 5-CQA also was shown to alleviate hepatic ischemia and reperfusion-induced liver injury by reducing inflammatory responses and increasing antioxidant defense systems [93]. There is also evidence that 5-CQA can suppress IL-1β, TNF-α, and IL-6 production in CCl4-induced liver inflammation and fibrosis in rats by inhibition of NF-κB activation [94]. Table 3 is a summary of cell and animal studies on the anti-inflammatory activity of CGA.

**Table 3:** Summary of studies that evaluated the capacity of CGAs to modulate inflammatory stress in cell-based and animal-based models.

Models	End-Point Measure	CGA Isomer	Concentration/Exposure Time	Results Compared to the Control without CGA Treatment	References
**Cell Models**					
Caco-2 + TNF-α and H_2_O_2_	IL-8	5-CQA	0.5–2 mM	↓ IL-8	[86]
Caco-2 + cocktail of inflammatory mediators	IL-6, IL-8	Mixture of all CGAs	Unknown composition of CGAs for 1 h	↓ IL-6, ↓ IL-8	[30]
RAW 264.7 + LPS	NO, IL-1β, TNF-α, cyclooxygenase-2, NF-κB, IL-6	5-CQA	2–20 μM for 24 h	↓ NO, ↓ IL-1β, ↓ TNF-α, ↓ IL-6, ↓ cyclooxygenase-2, ↓ NFκB	[87]
RAW 264.7 + LPS	Cyclooxygenase, Prostaglandin E2, NF-κB	5-CQA	12.5–37.4 μg/mL for 2 h	↓ Cyclooxygenase, ↓ Prostaglandin E2, ↓ NF-κB	[89]
**Animal Models**					
Trinitrobenzenesulfonic acid induced colitis mice model	Myeloperoxidase, H_2_O_2_, NF-κB	5-CQA	Orally administration at 20 mg/kg body weight, twice a day for 6 days	↓ Myeloperoxidase, ↓ H_2_O_2_, ↓ NF-κB	[48]
Dextran sulfate sodium induced colitis mice model	IL-1β, TNF-α, macrophage inflammatory protein 2	5-CQA	Orally administration at 1 mM for 15 days	↓ IL-1β, did not significantly change the levels of TNF-α and macrophage inflammatory protein 2	[86]
Trinitrobenzenesulfonic acid induced colitis mice model	Myeloperoxidase, H_2_O_2_, NF-κB	5-CQA	Orally administration at 20 mg/kg body weight twice a day	↓ Myeloperoxidase, ↓ H_2_O_2_, ↓ NF-κB	[90]
Rheumatoid Arthritis rat model	IL-1β, TNF-α, T cells count, Th1 cytokines, Th2 cytokines	5-CQA	Orally administration at 40 mg/kg body weight	↓ IL-1β, ↓ TNF-α, ↓ T cells count, ↓ Th1 cytokines, ↑ Th2 cytokines	[91]
Wounds in diabetic rat	Wound healing speed, NO, MDA, GSH	5-CQA	Intraperitoneal injection at 50 mg/kg/day for 15 days	↑ Wound healing speed, ↑ GSH, ↓ NO, ↓ MDA	[92]
Liver injury rat model	MDA, GSH, TNF-α, NO, cyclooxygenase-2 protein increase	5-CQA	Orally administration at 2.5–10 mg/kg body weight, twice a day	↓ MDA, ↑ GSH, ↓ TNF-α, ↓ NO, ↓ cyclooxygenase-2 protein increase	[93]

## 7. Summary and Conclusions

CGAs, which exist in green and roasted coffee beverages as well as fruit- and vegetable-based juices, are a group of esters formed between quinic acid and some trans-cinnamic acids, such as caffeic, ferulic, and *p*-coumaric acids. There has been considerable interest in understanding and quantitating the bioactivity of CGAs associated with antioxidant and anti-mutagenic activities related to oxidative stress. The topic is complex with the knowledge that CGAs are not stable in high temperature processed beverages, such as coffee [1,95]. This review highlights the cellular and molecular mechanisms that explain the pharmacological benefits of consuming CGA-containing beverages. Clearly, *in vitro* and *in vivo* data indicate that 5-CQA has antioxidant activity and can alleviate oxidative stress in various disease models. This potential pro-oxidant activity occurs under conditions that prolong the lifetime of the phenoxyl radical. The majority of the evidence to date indicates an anti-inflammatory activity for 5-CQA that can be explained by its ability to down regulate pro-inflammatory cytokines, through modulation of key transcription factors. A study reported that intravenous injection with 5-CQA at high dosages caused a range of inflammatory reactions in rats [96]. However, in all reality, the CGA dose infused did not relate to a typical dietary exposure. Future research is required to assess more of the potential health benefits of CGA-containing beverages. This should involve understanding the potential bioactivities of non-5-CQA isomers and the products of CGA transformation that occur in the large intestine from microbial activity.
